# Pro-Inflammatory Response to Macrotextured Silicone Implant Wear Debris

**DOI:** 10.1007/s11249-025-01965-6

**Published:** 2025-02-04

**Authors:** Dixon J. Atkins, Ann E. Rogers, Kathryn E. Shaffer, Ian Moore, Wyatt D. Miller, Meghan A. Morrissey, Angela A. Pitenis

**Affiliations:** 1 Interdisciplinary Program in Quantitative Biosciences, University of California, Santa Barbara, Santa Barbara, CA 93106, USA; 2 Molecular Cellular and Developmental Biology Department, University of California, Santa Barbara, Santa Barbara, CA 93106, USA; 3 Materials Department, University of California, Santa Barbara, Santa Barbara, CA 93106, USA; 4 Materials Research Laboratory, University of California, Santa Barbara, Santa Barbara, CA 93106, USA

**Keywords:** Biotribology, Friction, Silicone elastomers, Breast implants, Macrophages, Inflammation

## Abstract

Macrotextured silicone breast implants are associated with several complications, ranging from seromas and hematomas to the formation of a rare type of lymphoma, known as breast implant-associated anaplastic large cell lymphoma (BIA-ALCL). The presence of silicone wear debris has been detected within the peri-implant region and fibrotic capsule and histological analyses reveal inflammatory cells surrounding debris particles. However, it is unclear how these debris particles are generated and released from macrotextured implant surfaces, and whether wear debris generation is related to implant stiffness. In this study, we created an accelerated implant aging model to investigate the formation of silicone wear debris produced from self-mated (“shell-shell”) tribological interactions. We created implant-like silicone elastomers from polydimethylsiloxane (PDMS) using Sylgard 184 base:curing agent (10:1, 12:1, and 16:1) and quantified their mechanical properties (E* = 1141 ± 472, 336 ± 20, and 167 ± 53 kPa, respectively). We created macrotextured PDMS samples using the lost-salt technique and compared their self-mated friction coefficient (< *μ* > = 4.8 ± 3.2, 4.9 ± 1.8, and 6.0 ± 2.3, respectively) and frictional shear stress (*τ* = 3.1 ± 1.3, 3.2 ± 1.7, and 2.4 ± 1.4 MPa, respectively) to those of the recalled Allergan Biocell macrotextured implant shell (E* = 299 ± 8 kPa, < *μ* > = 2.2, and *τ* = 0.8 ± 0.1). Friction coefficient and frictional shear stress were largely insensitive to variations in elastic modulus for macrotextured PDMS samples and recalled implant shells. The stiffest 10:1 PDMS macrotextured sample and the recalled implant shell both generated similar area fractions of silicone wear debris. However, the recalled implant shell released far more particles (> 10×), mainly within the range of 5 to 20 μm^2^ in area. Bone marrow-derived macrophages (BMDMs) were treated with several concentrations of tribologically generated silicone wear debris. We observed widespread phagocytosis of wear debris particles and increasing secretion of inflammatory cytokines with increasing concentration of wear debris particles. Our investigation highlights the importance of avoiding macrotextured surfaces and mitigating wear debris generation from silicone implants to reduce chronic inflammation.

## 1 Introduction

Silicone breast implants have been widely used since the 1960s to improve form and function and accounted for 82% of cosmetic and reconstructive surgeries in 2021 [1]. Macrotextured silicone implants, defined as having an average surface roughness in excess of 50 μm were introduced in the 1980s with aggressively textured “Velcro”-like surfaces to mitigate malpositioning and complications like capsular contracture [1, 2]. Despite their widespread use, they largely failed to address these issues and created more severe complications. Since 2013, researchers have linked certain macrotextured breast implants with breast implant-associated anaplastic large cell lymphoma (BIA-ALCL), a rare cancer of the immune system, [3], with an average time to first diagnosis of about 8 years [4]. Mounting evidence led to the voluntary recall of Allergan Biocell in 2019 following request by the United States Food and Drug Administration (FDA) [5–7]. However, the rarity of severe complications has led the FDA to state that breast implants generally have “reasonable assurance of safety and efficacy” [8, 9] and do not recommend the prophylactic removal of macrotextured implants from asymptomatic patients [10]. The American Society of Plastic Surgeons estimates that the lifetime risk of BIA-ALCL is between 1:2207 (0.045%) and 1:86,029 (0.001%) for patients with macrotextured implants [11].

**Fig. 1:**
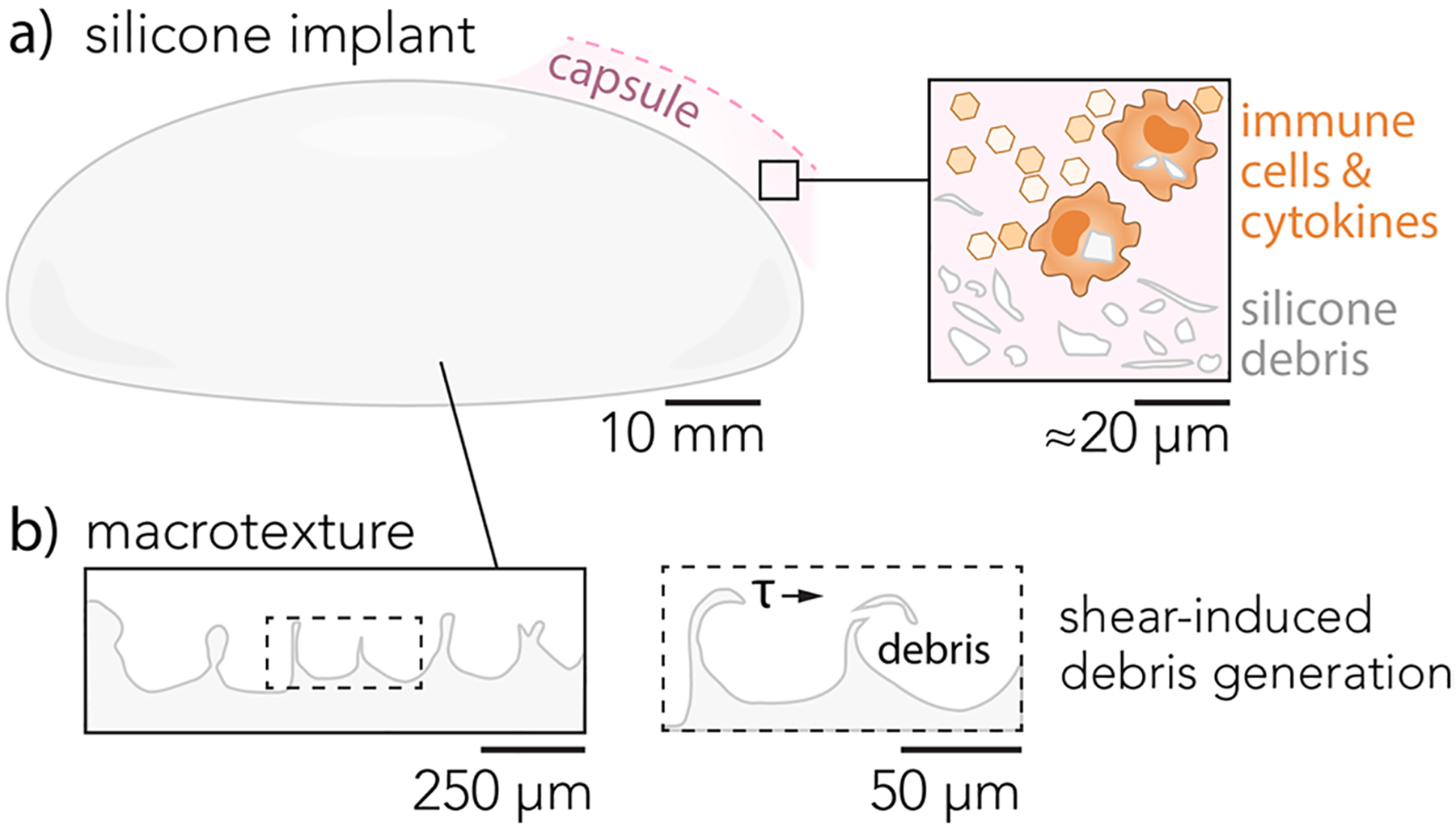
**a** Schematic of a macrotextured silicone breast implant shell and fibrotic capsule containing macrophages and silicone wear debris particles. **b** Cross-section of macrotextured silicone implant shell traced from scanning electron micrographs. The implant shell has an average surface roughness of approximately *R*_a_ ≈ 80 μm. Inset shows schematic of frictional shear stress, *τ*, acting on high aspect ratio surface features (≈10s μm width, ≈160 μm height) that may form wear debris and provoke inflammation

Although the precise etiology of complications including capsular contracture, breast implant illness, and BIA-ALCL remain poorly understood, several groups have implicated adverse tribological interactions between implants and surrounding tissue [12–15]. However, definitive relationships between surface roughness, friction, silicone wear debris generation, and inflammation have yet to be established for macrotextured silicone implants. Silicone debris particles accompanied by inflammatory large giant cells were detected within peri-implant capsules 6 months post-implantation in rabbit models [16]. In contrast, no silicone debris was detected in capsules surrounding smooth implants [16]. The quantity and morphology of debris particles released from breast implants are highly dependent on the implant type, and the effects of implant stiffness have been largely unexplored [17–19]. Immune cells in the fibrotic capsule may interact with silicone debris and the sizes and geometries of these particles drive inflammatory responses [20–23] (Fig. 1). Macrophages also show sensitivity to particle stiffness, and biophysical cues can determine whether disease states resolve or become chronic [24–26]. A chronic inflammatory environment is thought to facilitate malignancy in BIA-ALCL [27–29].

In this investigation, we prepared polydimethylsiloxane (PDMS) elastomers with macrotextured surfaces similar in surface profile and stiffness to the recalled Allergan Biocell implant shell. The PDMS base:curing agent ratio was varied (10:1, 12:1, and 16:1) to create samples that exhibited similar mechanical properties (e.g., elastic modulus) to the recalled implant shell. PDMS samples were used in self-mated friction measurements to model tribomechanical microtraumas that may occur over the lifetime of the implant (e.g., wrinkling, folding). Silicone wear debris particles were collected following tribological experiments and added to in vitro culture of mouse bone marrow-derived macrophages (BMDMs) [30]. We used fluorescence imaging and cytokine bead arrays to investigate the extent of macrophage pro-inflammatory cytokine secretion in response to tribologically generated silicone wear debris.

## 2 Materials and Methods

### 2.1 PDMS Sample Preparation and Characterization

Polydimethylsiloxane (PDMS) samples were prepared using Sylgard 184 by mixing the silicone base material with its cross-linker curing agent in ratios of 10:1, 12:1, and 16:1 base:curing agent, determined by weight. A dilute concentration (0.005 vol.%) of 26 nm diameter green fluorescent beads (Thermo Scientific, Cat. No. G25, excitation/emission: 468/508 nm) was added to PDMS samples for imaging. The base, curing agent, and fluorescent beads were mixed using a FlackTek SpeedMixer (model DAC 150.1 FV-K) for 1 min at 2,500 × *g* and placed into a polystyrene dish for a final sample thickness of 0.5 mm to match the thickness of textured implant samples. PDMS samples were degassed in a vacuum oven at 50 °C for 10 min. Smooth PDMS samples were cured at 90 °C for 24 h. Macrotextured PDMS samples had salt crystals (NaCl, Millipore Sigma, Cat. No. 7710–500GM, approximately 180 μm in diameter) added to the surface of the PDMS, following the methods in Barr et al. [31] before curing at 90 °C for 24 h. Macrotextured PDMS samples were sonicated three times in ultrapure water to remove salt crystals. Cross-sections of the textured PDMS and implant surfaces were examined using scanning electron microscopy (Supplementary Fig. S1). Infrared spectroscopy (O-PTIR, MRL shared facilities) of the smooth surfaces of PDMS elastomers were compared to the reverse (smooth) side of the implant and similar spectra and peaks were observed (Supplementary Fig. S2) [32]. Hydrogel probes with attached PDMS sections and PDMS countersurfaces (or implant) were prepared, previously described in Atkins et al. [33].

### 2.2 Indentation Measurements Against Smooth Elastomers

The reduced elastic modulus, E*, of smooth PDMS samples were determined using microindentation techniques previously described in Chau et al. [34]. Briefly, PDMS substrates were indented with a hemispherical borosilicate glass probe with a radius of curvature, *R* = 2.6 mm, mounted to a double-leaf cantilever flexure with a normal stiffness of *K*_*n*_ = 230 μN/μm. The reduced elastic modulus, E*, was determined using Hertzian contacts mechanics theory (Eq. 1) fit to approach curves at a normal load of *F*_*n*_ = 1 mN, probe radius of curvature, *R*, and indentation depth, *d*. The elastic moduli reported for PDMS substrates are an average and standard deviation of 3 individual samples across 5 indents of the same location per sample. The elastic modulus reported for the implant sample is the average and standard deviation of 5 indents across the same location.


(1) $$F_{n}=\frac{4}{3}E^{*}R^{\frac{1}{2}}d^{\frac{3}{2}}$$

### 2.3 Contact Area Measurements of Macrotextured Elastomers

Nominal contact area measurements of textured PDMS samples were made using a sphere-on-flat tribological testing configuration similar to the one described in Sect. 2.1 above. Hemispherical probes of macrotextured PDMS indented a flat glass plate (0.2 mm thickness) using the custom-built linear reciprocating tribometer previously described in Urueña et al. [35] attached to the condenser lens turret of a Nikon AR1 HD Ti2 confocal inverted microscope. Normal forces were measured using a titanium double-leaf cantilever flexure with normal stiffness, *K*_*n*_ = 200 μN/μm, and a capacitance probe mounted near the flexure in the normal direction (Lion Precision, model no. CPL190, 5 μm/V sensitivity, 200 μm range). Fluorescent textured PDMS probes were brought into contact with the glass plate at loads of 1 mN, 3 mN, and 5 mN (*n* = 3 per normal force) and imaged at the glass plane of contact with a 4× microscope objective (NA = 0.13). Contact areas were quantified using implant shell autofluorescence. Raw images were processed in FIJI by adjusting image intensity to a range of 0–1500, thresholding from 0 to 250, and using the “analyze particles” function to quantify the area of contact (see example in Supplementary Fig. S3). The mean and standard deviation are reported for *n* = 3 samples per PDMS or implant shell configuration for *n* = 3 normal loads.

### 2.4 Friction Measurements of Macrotextured Elastomers

Friction measurements were conducted using another custom linear reciprocating tribometer, previously described in [36, 37]. A double-leaf cantilever flexure with a normal stiffness of *K*_*n*_ = 230 μN/μm and *K*_*t*_ = 90 μN/μm was used with capacitance probes mounted in the normal and tangential directions (Lion Precision, model no. CPL190, 5 μm/V sensitivity, 200 μm range). Hydrogel probes with attached PDMS sections, previously described in [33, 37] were placed in a self-mated PDMS-PDMS orientation. The path length, *l*, (1/2 cycle) was 3 mm, and 1000 cycles of sliding were performed at a speed *v* = 0.5 mm/s under a targeted load of *F*_*n*_ = 1 mN to generate debris particles that may form over a decade of wear at the implant interface. Wear debris was collected and stored in ultrapure water before optical analysis.

### 2.5 Scanning Electron Microscopy of Macrotextured Elastomers and Wear Debris

Cross-sections of macrotextured PDMS and implant shells were imaged using scanning electron microscopy (SEM) (Supplementary Fig. S1). Silicone wear debris collected from friction experiments were secured to an SEM sample mount using adhesive carbon tape. To mitigate charging effects during SEM, samples were coated in a thin gold film (≈10 nm thick) using a SuPro Instruments ISC 150 T HV Ion Sputter Coater before SEM imaging (Thermo Scientific Apreo C LoVac SEM) in the Microscopy and Microanalysis Facility at UC Santa Barbara.

### 2.6 Culture of Mouse Bone Marrow-Derived Macrophages (BMDMs)

Bone marrow-derived macrophages were generated from female and male C57BL/6 mice between 6 and 10 weeks as described previously [30]. Briefly, bone marrow cells were harvested from femurs and cultured in RPMI-1640 supplemented with 10% fetal bovine serum, 1% penicillin–streptomycin-glutamine, and 20% conditioned media from L-929 MCSF-secreting cells (ATCC; CCL1) for 7 days. Differentiation was confirmed with flow cytometry via CD11b and F4/80 signal. Differentiated macrophages were used in experiments between days 7 and 12.

### 2.7 Confocal Imaging and Analysis of BMDMs and Macrotextured PDMS Wear Debris

Macrophages were cultured in a 96-well plate and imaged immediately following treatment with silicone wear debris particles. Timelapse images were taken across 9 positions per well every 15 min for 24 h. The plate stimulated for cytokine analysis downstream had still images captured at 0 h and 24 h. All images were acquired using a 40× air objective (NA = 0.95) on a Nikon Ti2-E inverted microscope containing an Orca Fusion BT scMos camera and Yokogawa CSU-W1 spinning disk unit. Confocal microscopy images of green-fluorescently labeled PDMS wear debris were counted manually using the geometric selection tool in FIJI and aggregated. Percent area of wear debris particles was calculated as an average over 18 unique 370 × 370 μm^2^ fields of view.

### 2.8 Brightfield Imaging and Analysis of Macrotextured Implant Shell Wear Debris

Brightfield images of implant debris particles were acquired using a Nikon Ti2-E Widefield Fluorescence Microscope and a 40× air objective (NA = 0.95). The distribution of debris particles was acquired from a total of 9 fields of view with 370 × 370 μm^2^ dimensions. Particle counts were generated using a threshold on raw brightfield images of 0–27,372 and excluding particles below the detection threshold, 0.50 μm^2^.

### 2.9 Macrophage Cytokine Bead Array

BMDMs were plated in a 96-well plate at 50,000 cells per well in culture medium. After 24 h, 20 μL of phosphate buffered saline (PBS 1X, negative control) or 20 μL of silicone wear debris suspended in PBS was added to cells. The concentration of silicone wear debris was either concentrated or diluted to achieve a range of particle area fraction in each well between 0 and 1%. Lipopolysaccharide (LPS), a bacterial cell membrane component known to provoke pro-inflammatory responses in macrophages, was added to positive control wells at a final concentration of 50 nM. After 24 h, 25 μL of culture media was sampled from each well and analyzed for cytokine secretion (IL-6, IL-10, MCP-1, IFN-y, TNF, and IL-12p70) using the BD Mouse Inflammation Kit (cat no. 552364) according to the manufacturer’s instructions, with volumes uniformly reduced by half. Cytokine bead array samples were examined using the Attune NxT (Invitrogen) and analyzed with FlowJo to determine the median fluorescence intensity of each cytokine.

## 3 Results and Discussion

### 3.1 Microindentation Results of Smooth Elastomers

**Fig. 2:**
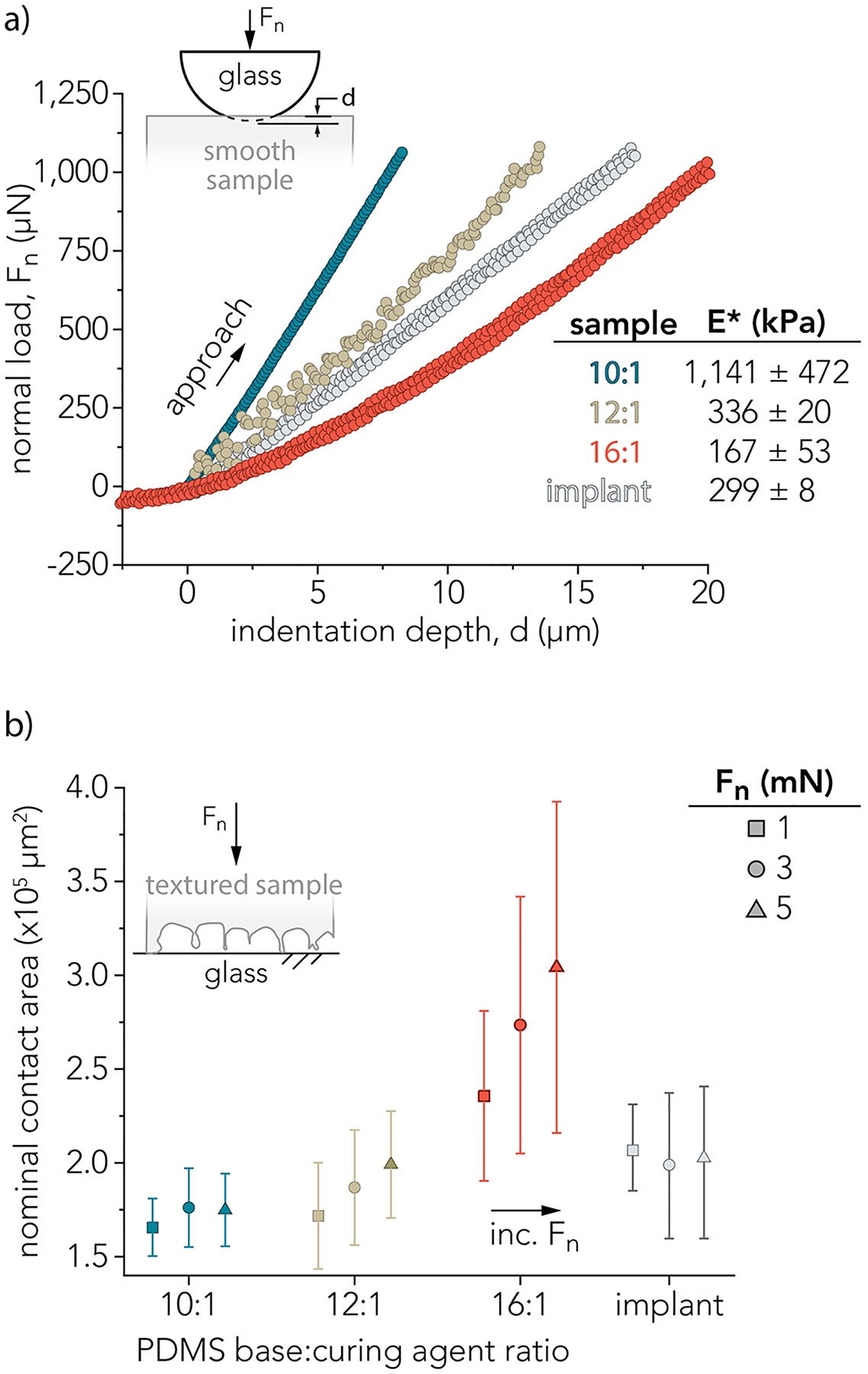
**a** Indentation measurements of smooth PDMS surfaces. **b** In situ optical characterization of textured PDMS surfaces indenting flat glass disks give nominal contact area for 3 different PDMS crosslinking ratios: 10:1; 12:1; and 16:1 (base:curing agent) at 3 different normal forces, *F*_*n*_ = 1 mN (squares), 3 mN (circles), and 5 mN (triangles). One textured implant surface was also characterized. The error bars represent the standard deviation over *n* = 3 measurements

To evaluate the role of elastic modulus in creating silicone wear debris, microindentation measurements were performed on smooth PDMS surfaces with elastic moduli above and below that of the smooth (reverse) surface of the recalled implant shell. Figure 2a shows approach curves generated using microindentation. The microindenter was equipped with a borosilicate hemispherical probe, and samples approximately 0.5 mm thick were indented to a normal load *F*_*n*_ = 1 mN at a velocity of 10 μm/s. The Hertz model was used to fit these approach curves and extract a reduced elastic modulus, E*, as described in Eq. 1. The table in Fig. 2a shows the average and standard deviation across at least 3 samples per PDMS base:curing agent ratio and 5 indents per sample. The average and standard deviation of one implant sample with 5 indents on this sample are reported as well. The average E* values were 1,141 ± 472, 336 ± 20, and 167 ± 53 kPa for the 10:1, 12:1, and 16:1 PDMS, respectively. The reduced elastic modulus of the reverse side of the implant shell was E* = 299 ± 8 kPa.

### 3.2 Contact Area of Macrotextured Surfaces Generally Increases with Normal Load

Contact area was evaluated for macrotextured surfaces in static contact with smooth and flat glass plates. Figure 2b shows the nominal area of contact measured by fluorescence intensity from confocal microscopy over 3 different normal loads (*F*_*n*_ = 1, 3, and 5 mN). Implant shell autofluorescence was leveraged to characterize contact area. While the fluorescence intensity was significantly lower than the macrotextured PDMS samples and contact area measurements may be underestimated, there is generally good agreement between the reduced elastic modulus, E*, and the nominal contact area between the 12:1 PDMS and the implant shell. The contact area generally increased with decreasing E* and increasing normal load, *F*_*n*_.

### 3.3 Tribological Measurements of Macrotextured Elastomers

**Fig. 3:**
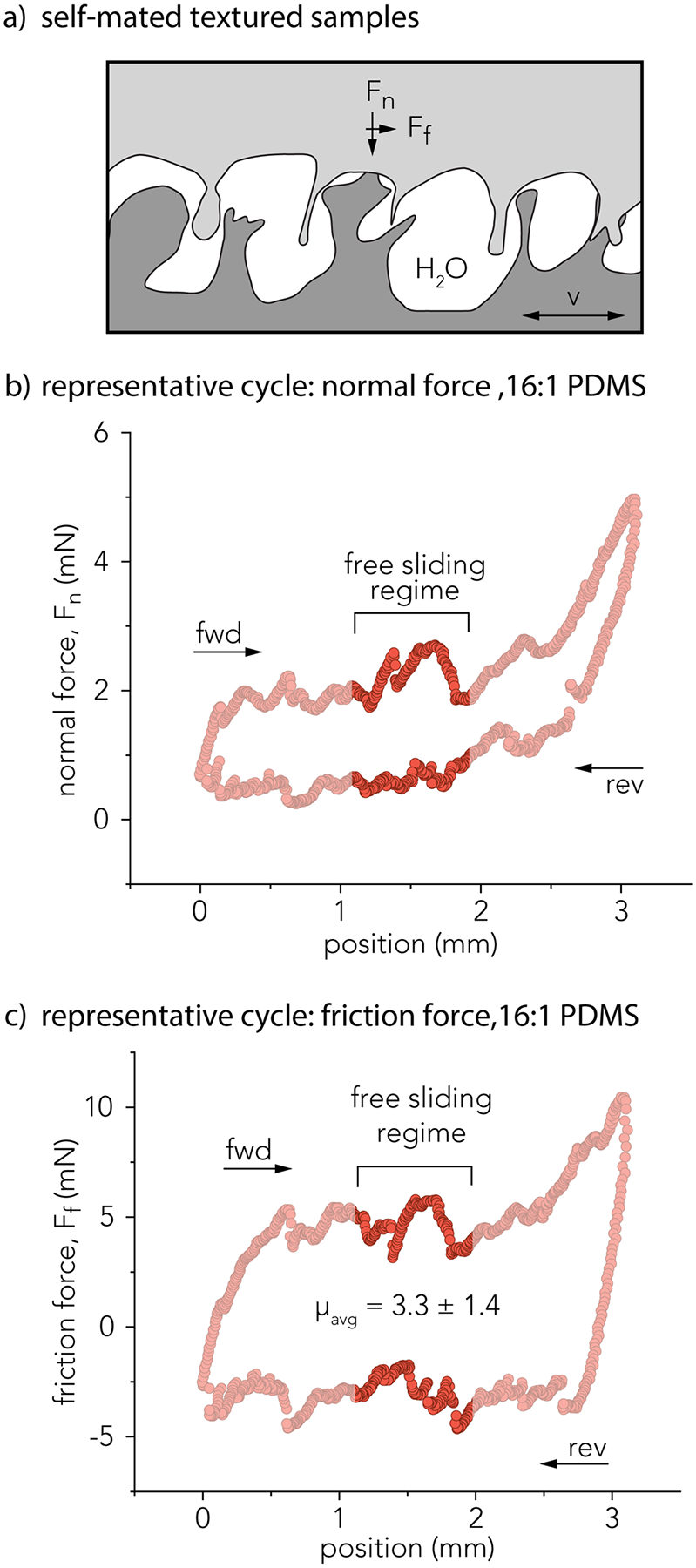
Friction measurements of self-mated textured PDMS surfaces. **a** Schematic of experimental configuration. **b** Representative normal force, *F*_*n*_ (mN) and **c** friction force, *F*_*f*_ (mN) as a function of sliding position (mm) for a single experiment (16:1 PDMS)

Figure 3a shows a schematic of the self-mated textured samples involved in friction measurements. All sliding experiments were performed with the countersurface and the probe fully submerged in ultrapure water. Each experiment was conducted for 1,000 reciprocating cycles over (6 m of total sliding distance) under a targeted normal force of *F*_*n*_ = 1 mN and a constant velocity of sliding, *v* = 0.5 mm/s. The high surface roughness of these silicone surfaces presented challenges associated with maintaining constant normal force. Figure 3b shows the representative normal force across a single reciprocating cycle from the self-mated 16:1 PDMS macrotextured surfaces, and Fig. 3c shows the corresponding friction force. The average friction coefficient for all sliding cycles was analyzed across the free sliding regime, within the middle 600 μm of the sliding path and away from the reversals. The average and standard deviation of the friction coefficient for this representative sliding cycle is reported in Fig. 3c, *μ*_avg_ = 3.3 ± 1.4.

**Fig. 4:**
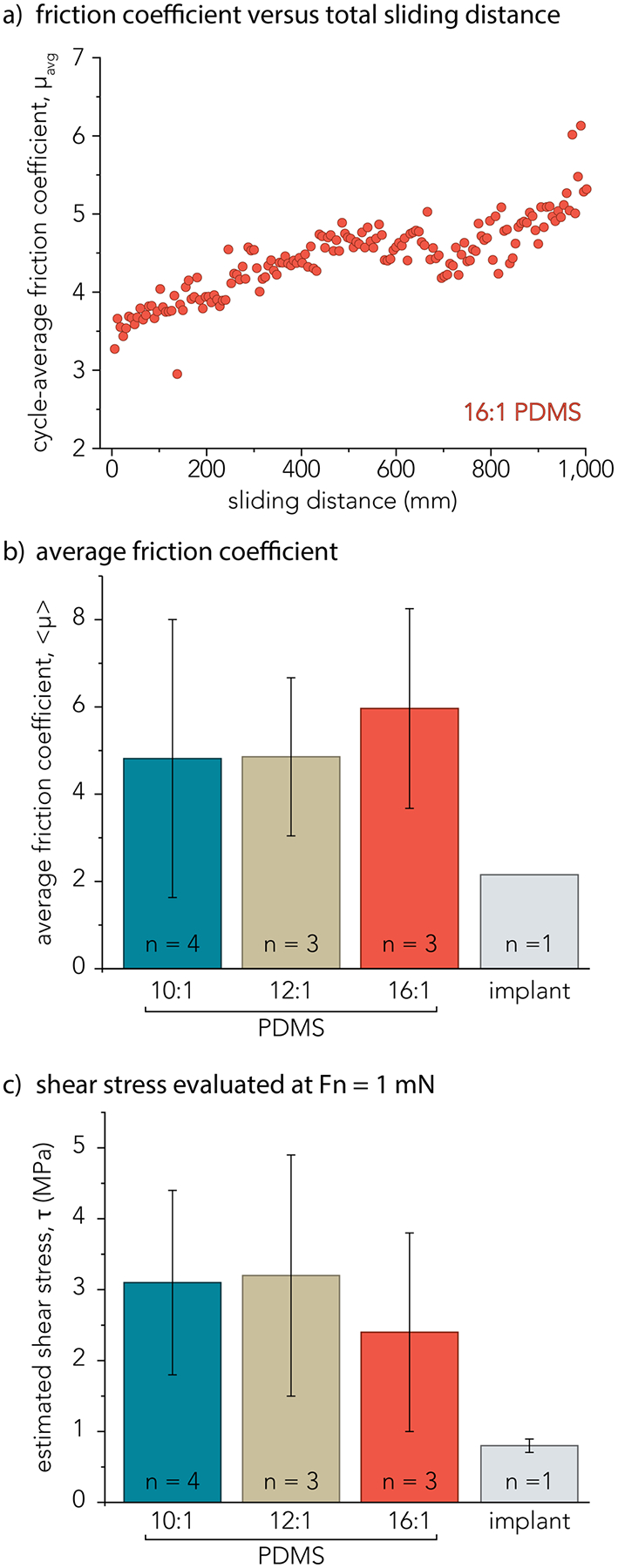
**a** Representative friction coefficient data for each cycle, *μ*_avg_, plotted versus sliding distance for a single continuous experiment for a 16:1 PDMS sample (*n* = 1). **b** Bar plot showing the experiment-averaged friction coefficient, <*μ*> for *n* = 3 experiments for PDMS samples and *n* = 1 experiment for the implant sample. Error bars represent the standard deviation in experiment-averaged friction coefficients. **c** Bar plot of the frictional shear stress, *τ*, estimated for each PDMS sample and implant sample from static indentation measurements at *F*_*n*_ = 1 mN. Error bars represent the combined uncertainty of frictional shear stress measurements, *u(τ)*

The friction coefficient for each reciprocating cycle is *μ*_*avg*_, which is reported as a single data point in Fig. 4a. The average of all points in Fig. 4a and across n = 3 sliding experiments is reported as the experiment-averaged friction coefficient, < *μ* > = 6.0 ± 2.3. Representative *μ*_*avg*_ data as a function of sliding distance for the 16:1 PDMS self-mated friction measurement is displayed in Fig. 4a. Representative *μ*_avg_ data with 10:1 and 12:1 PDMS samples are located in Supplementary Figure S4. The experiment-averaged friction coefficient, < *μ* >, for each sample are reported as a bar plot in Fig. 4b. The average friction coefficient across 1 sliding experiment over 1000 mm of sliding (n = 1) is reported for the implant sample and is in accordance with prior literature values [33].. There are no statistical differences between < *μ* >; the average friction coefficient for PDMS samples in self-mated contact may be more sensitive to the high surface roughness than to elastic modulus despite ranging nearly an order of magnitude. The average friction coefficient as a function of sliding distance across at least *n* = 3 experiments can be found in Supplementary Figure S5.

The average shear stress, *τ*, was estimated for each macrotextured elastomer in Fig. 4c using Eq. 2, below, where *F*_*f*_ is the average friction force and *A*_contact_ is the nominal area of contact at *F*_*n*_ = 1 mN.


(2) $$\tau =\frac{F_{f}}{A_{\text{contact}}}$$

The average friction force, *F*_*f*_, was measured across at least n = 3 experiments for each macrotextured PDMS elastomer and for n = 1 for the recalled implant shell. Estimates of the frictional shear stress, *τ*, are approximately 3 – 10 times greater than the reduced elastic modulus, E*, of these samples and are roughly half the reported breaking stress of macrotextured silicone breast implants (6 MPa) [38]. Error bars in Fig. 4c reflect the combined uncertainty in shear stress measurements, *u*(*τ*), calculated using Eq. 3 below, where *u*(*F*_*f*_) is the standard deviation in friction force and *u*(*A*_contact_) is the standard deviation of the nominal contact area.


(3) $$u\left(\tau \right)=\sqrt{{\left(\left(\frac{1}{A_{\text{contact}}}\right)*u\left(F_{f}\right)\right)}^{2}+{\left(-\left(\frac{F_{f}}{A_{\text{contact}}^{2}}\right)*u\left(A_{\text{contact}}\right)\right)}^{2}}$$

### 3.4 Quantifying Wear Debris

**Fig. 5:**
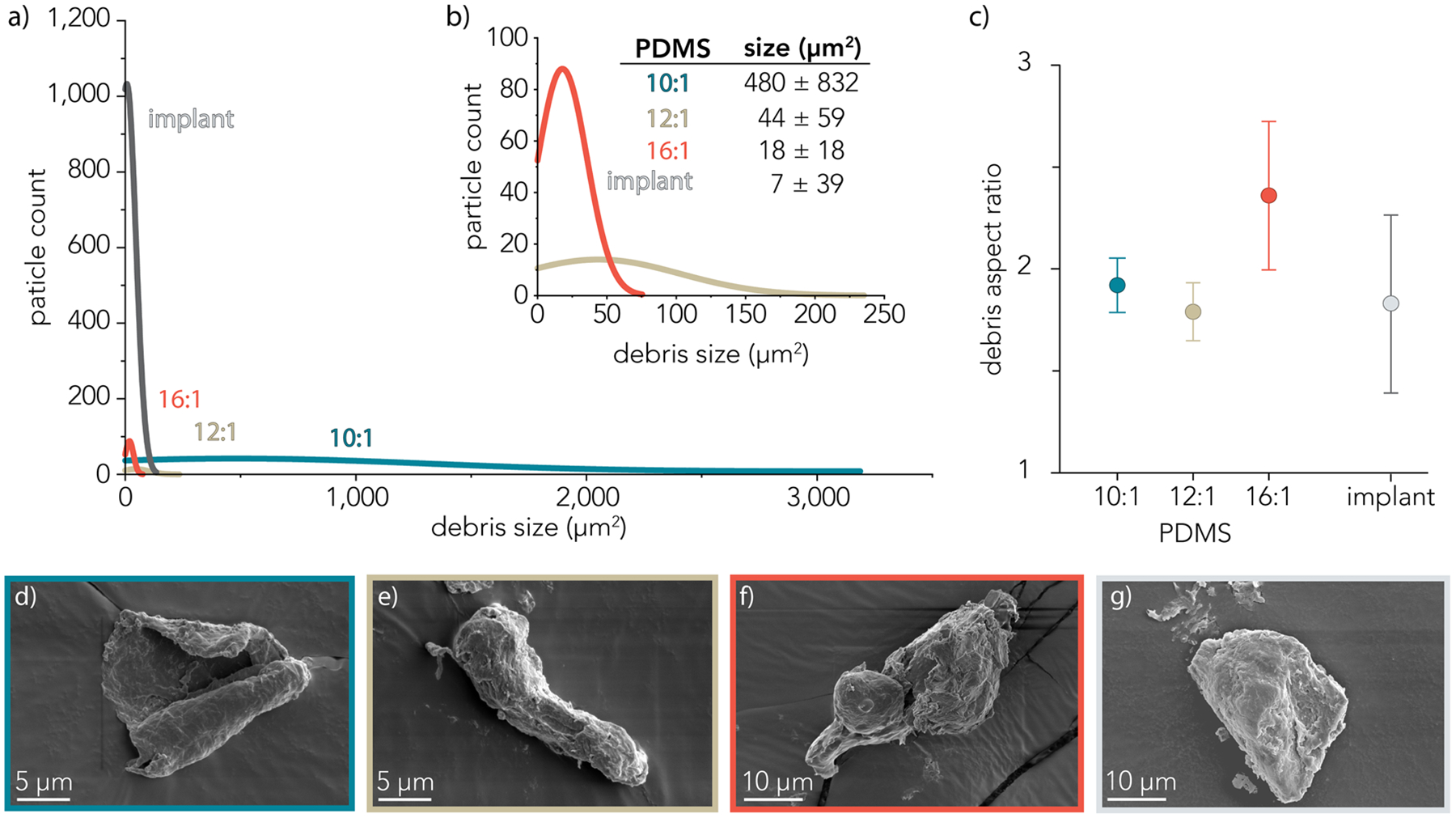
**a** Particle size distribution for wear debris released following friction measurements using three sample formulations: 10:1; 12:1; 16:1 PDMS (*n* = 3 independent friction experiments) and *n* = 1 implant sample. **b** Inset of **a** showing particle size distribution of 12:1 and 16:1 PDMS formulations. **c** Aspect ratio of debris particles analyzed from (**a**) for 10:1, 12:1, and 16:1 PDMS sample formulations and 1 implant sample. Scanning electron micrographs of representative debris particles from **d** 10:1, **e** 12:1, **f** 16:1 PDMS, and **g** implant sample friction measurements

Figure 5a shows distribution curves of wear debris generated over sliding experiments for 10:1, 12:1, and 16:1 PDMS and one implant sample. Both the 10:1 PDMS and implant sample generated a similar total area of debris, but the 10:1 PDMS samples generated some very large debris particles, and the implant sample generated large amounts of small particles. Figure 5b shows an inset of part (a) with the particle distributions of the 12:1 and 16:1 PDMS samples. The average size of wear debris particles were measured as 480 ± 823 μm^2^, 44 ± 59 μm^2^, 18 ± 18 μm^2^, and 7 ± 39 μm^2^ for the 10:1, 12:1, 16:1, and implant samples, respectively. The distribution of PDMS debris particles was measured using fluorescently tagged PDMS samples (examples shown in Supplementary Figure S6), and implant wear debris was characterized using brightfield imaging. Figure 5c displays the aspect ratio of the measured debris particles, and it shows that the 16:1 PDMS wear debris particles have a modestly higher aspect ratio relative to wear debris from other PDMS formulations or implant shell samples. Figure 5d–g show representative SEM images of wear debris particles from (d) 10:1 PDMS, (e) 12:1 PDMS, (f) 16:1 PDMS, and (g) the implant sample and highlight the irregular morphologies formed from friction and fracture.

### 3.5 Immune Response to Silicone Debris

**Fig. 6:**
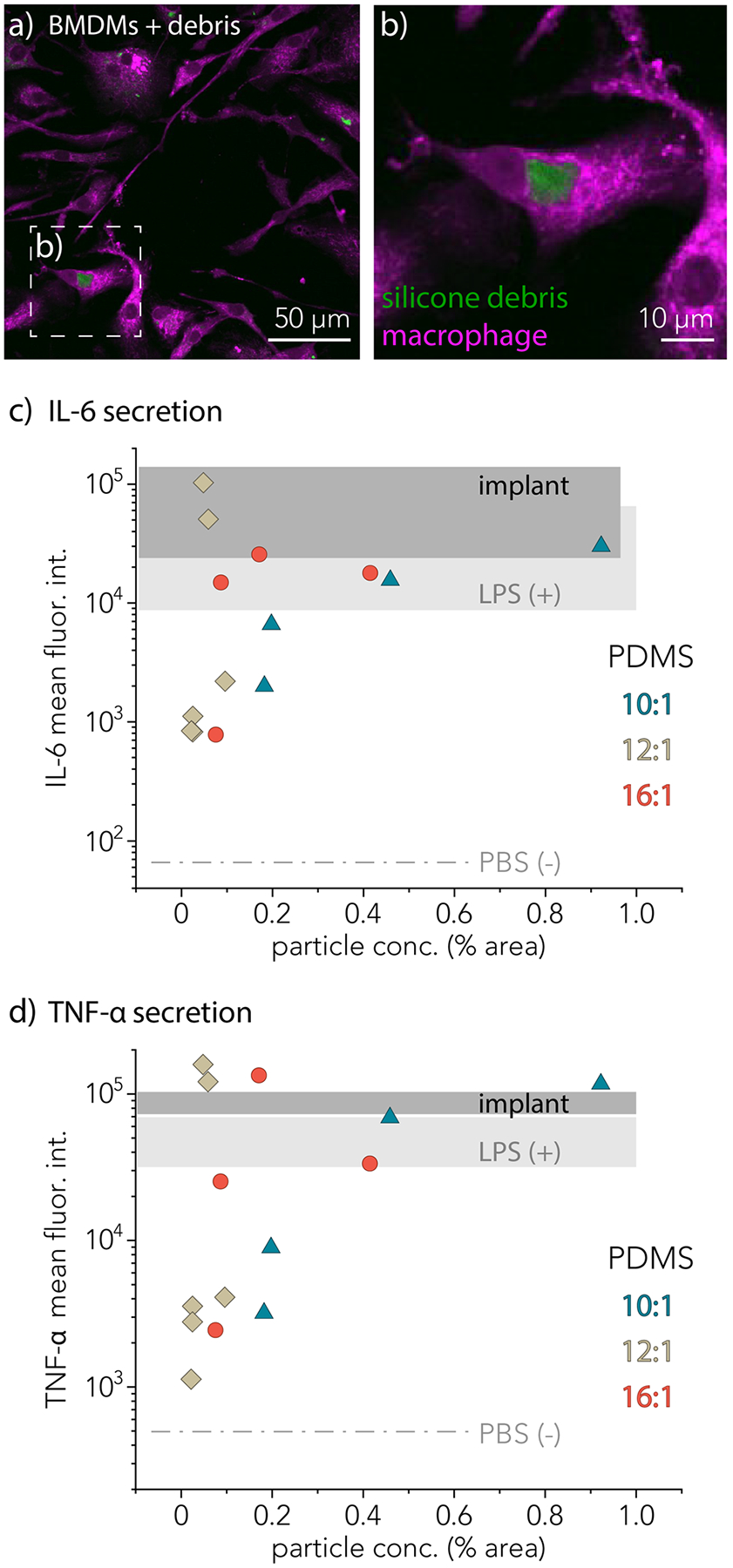
**a** Representative confocal image showing phagocytosed debris by mouse bone-marrow-derived macrophages. **b** Inset of **a** showing detail of phagocytosed silicone debris particle. Mean fluorescence intensity of secreted **c** IL-6 and **d** TNF-α as a function of seeding concentration of debris particles (area %), which were collected as an average percent area over 18 unique fields of view (370 × 370 μm) per sample. Each data point represents the average cytokine secretion from one well of macrophages plated in a 96-well plate

To investigate the extent to which immune cells interact with silicone debris generated from self-mated sliding contact, we dosed concentrations of silicone debris into a 96-well plate each containing about 50,000 mouse bone marrow-derived macrophages (BMDMs). Figure 6a shows a confocal image of a field of view containing several macrophages and fluorescent silicone debris particles. The macrophages clearly phagocytose silicone debris particles, shown in Fig. 6a and b. Figure 6b highlights a silicone wear debris particle (10:1 PDMS) engulfed by a BMDM.

To test the secretion of inflammatory cytokines by BMDMs in the presence of phagocytosed silicone wear debris particles, a cytokine bead array was performed. Each of the points on Fig. 6c and d represents the quantity of inflammatory cytokine secreted at a controlled area percentage of silicone wear debris quantified using confocal microscopy. Figure 6c shows the amount of inflammatory cytokine IL-6 secreted in the media. The negative control, PBS, is shown as a dashed line, and the positive control, LPS, is shown as a shaded region in light gray. All points in this LPS region are similar to the positive control and produce a significant amount of IL-6. BMDMs treated with wear debris from the implant sample also secreted amounts of IL-6 similar to the positive control. Figure 6d shows the secretion of inflammatory cytokine TNF-α. The negative control, PBS is shown as a dashed line, and the positive control, LPS, is shown as a gray shaded region. Remarkably, wear debris generated from the implant sample caused TNF-α secretion far greater than the positive control. Some concentrations of wear debris particles from all PDMS elastomers exceeded the positive control.

Our results suggest a dependence on wear debris particle concentration on cytokine secretion in BMDMs. In general, lower debris concentrations are less likely to induce secretion of either IL-6 or TNF-α. However, we observed that even very low concentrations (< 0.1% area) of 12:1 and 16:1 PDMS wear debris particles induced cytokine secretion in BMDMs. We hypothesize that this may be a result of macrophages interacting with a greater number of small particles. Another explanation is that wear debris particles formed from the 16:1 PDMS exhibited modestly higher aspect ratios, which are correlated with pro-inflammatory macrophage responses [39]. The 10:1 PDMS wear debris provoked dose-dependent inflammatory responses, although macrophages may have been disproportionately affected by few very large particles (Fig. 5a,b). Additionally, some macrophages treated with large amounts of silicone wear debris exhibited greater binucleation, potentially related to a disturbance of the cell cycle or interactions with IL-6 (Supplementary Figure S7) [40]. Particle stiffness may also play a role in macrophage cytokine secretion [25, 26, 41]. We cultured BMDMs with soft (E* ≈ 1 kPa) polyacrylamide particles of similar aspect ratio, size, and morphology (Supplementary Figure S8), but we observed no inflammatory cytokine secretion (Supplementary Figure S9). These results suggest that macrophages are more sensitive to wear debris particles of certain ranges in elastic modulus.

## 4 Concluding Remarks

In this investigation, we developed a new in vitro platform to accelerate aging in breast implant samples using controlled geometries and tribological challenges. We examined the tendency of macrotextured silicone implant-like surfaces to generate and release wear debris and compared these results with a recalled breast implant shell (Allergan Biocell). This platform enabled investigations into the relationship between the elastic modulus of macrotextured elastomers and wear debris generation in self-mated sliding. The friction coefficient across self-mated macrotextured elastomers was largely driven by the very high surface roughness, with no statistical differences in average friction coefficients, < *μ* >. The softest sample, the 16:1 PDMS elastomer, exhibited the highest contact area although the frictional shear stress did not differ significantly from other samples. The frictional shear stress for all elastomers were far in excess of the elastic modulus and nearly half the reported breaking stress of PDMS, 6 MPa [38], indicating high likelihood of fracture and wear debris generation during sliding. We observed a large quantity (area %) of wear debris produced following self-mated 10:1 PDMS and recalled implant sliding experiments compared to 12:1 and 16:1 PDMS. The 10:1 PDMS wear debris was the largest in size and distribution, and the recalled implant shell generated the smallest particles and the greatest number of wear debris particles. We cultured mouse bone marrow-derived macrophages with silicone wear debris generated from self-mated friction experiments and discovered that increasing concentration of particles increased the secretion of strong pro-inflammatory cytokines, IL-6 and TNF-α. Our results suggest that increasing surface roughness is the primary mechanism of wear debris generation in silicone implants. Wear behavior of macrotextured elastomers does not strongly benefit from reducing the elastic modulus. Since many breast implant related complications, including capsular contracture and BIA-ALCL are progressive and may develop slowly over many years, wear debris generation over time must be included in future implant designs. The informed design of silicone implant surfaces is essential for limiting the accumulation of non-digestible wear debris particles that can stimulate the secretion of inflammatory cytokines and provoke chronic disease.

## Supplementary Material

Supplement

## Data Availability

The data supporting the findings in the manuscript are located in the Supplementary Materials and are freely available in Data Dryad at https://doi.org/10.5061/dryad.m37pvmdcw.
