# Supplementary material for: Pro-Inflammatory Response to Macrotextured Silicone Implant Wear Debris
Source: Tribol Lett. Author manuscript; Available in PMC 2025 Jul 31. (PMC12313313; doi:10.1007/s11249-025-01965-6)
Supplement: Supplement [file NIHMS2063021-supplement-Supplement.docx]

**Supplementary Information for:**

**Pro-Inflammatory Responses to Silicone Implant Wear Debris**

Dixon J. Atkins^1^, Ann K. Rogers^2^, Kathryn Shaffer^3^, Ian Moore^4^, Wyatt D. Miller^1^, Meghan A. Morrissey^2^, and Angela A. Pitenis^3,4^

^1^Interdisciplinary Program in Quantitative Biosciences

University of California, Santa Barbara

Santa Barbara, California, 93106, USA

^2^Molecular Cellular and Developmental Biology Department

University of California, Santa Barbara

Santa Barbara, California, 93106, USA

^3^Materials Department

University of California, Santa Barbara

Santa Barbara, California, 93106, USA

^4^Materials Research Laboratory

University of California, Santa Barbara

Santa Barbara, California, 93106, USA

**Corresponding Author**

Angela A. Pitenis

Associate Professor

Materials Department

University of California, Santa Barbara

Santa Barbara CA 93106

email: apitenis@ucsb.edu


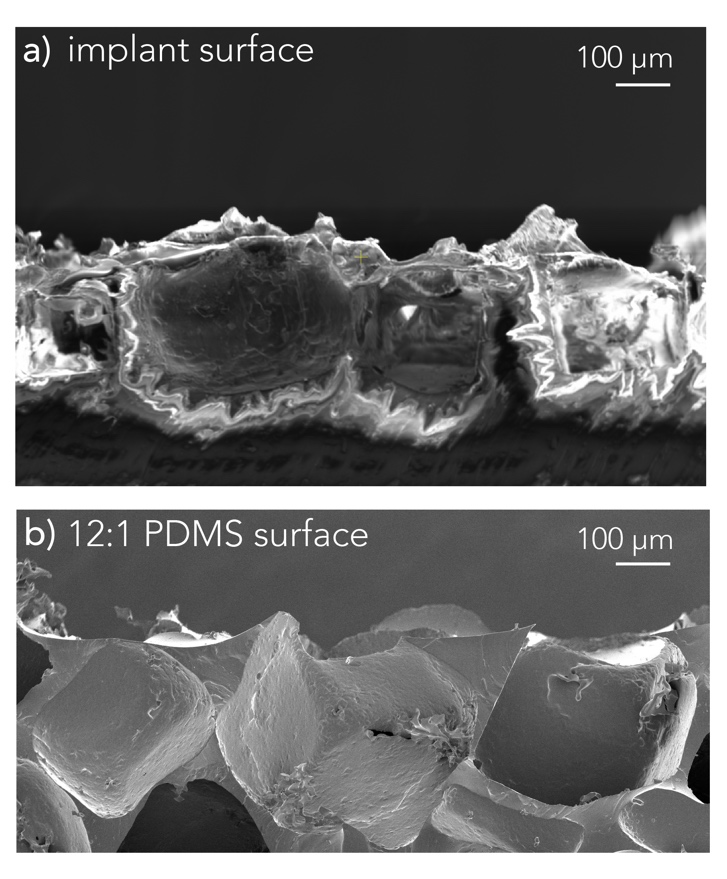


**Fig. S1.** Scanning electron micrograph cross-sections of (a) implant surface and (b) 12:1 PDMS surface.


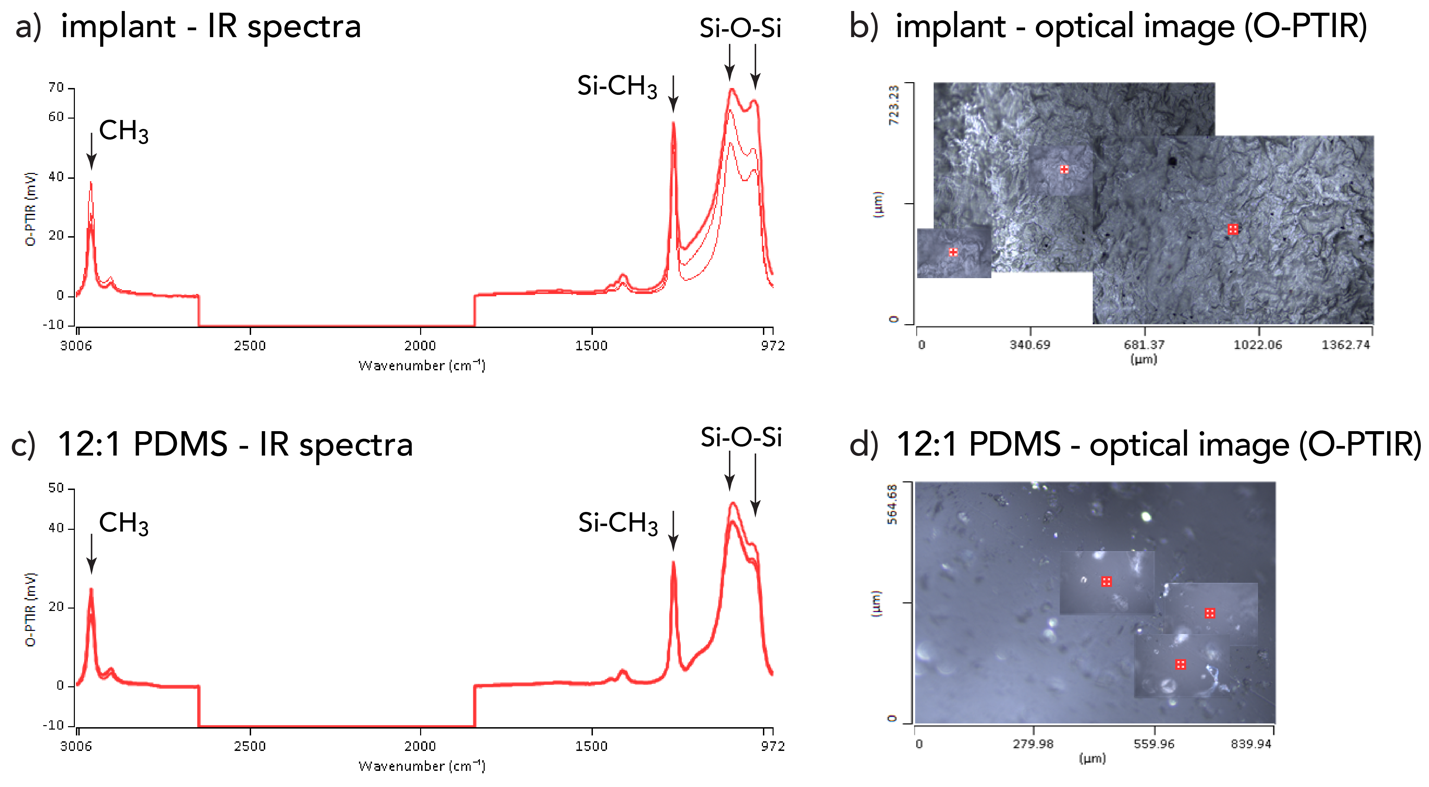


**Fig. S2.** Infrared spectroscopy spectra of smooth surface of textured (a) implant and (c) 12:1 PDMS with labeled CH_3_, Si-CH_3_, and Si-O-Si peaks. (b, d) Optical images of regions selected for IR spectroscopic analysis using an optical photothermal infrared microscope (O-PTIR, mIRage® IR microscope, Photothermal Spectroscopy Corp.).


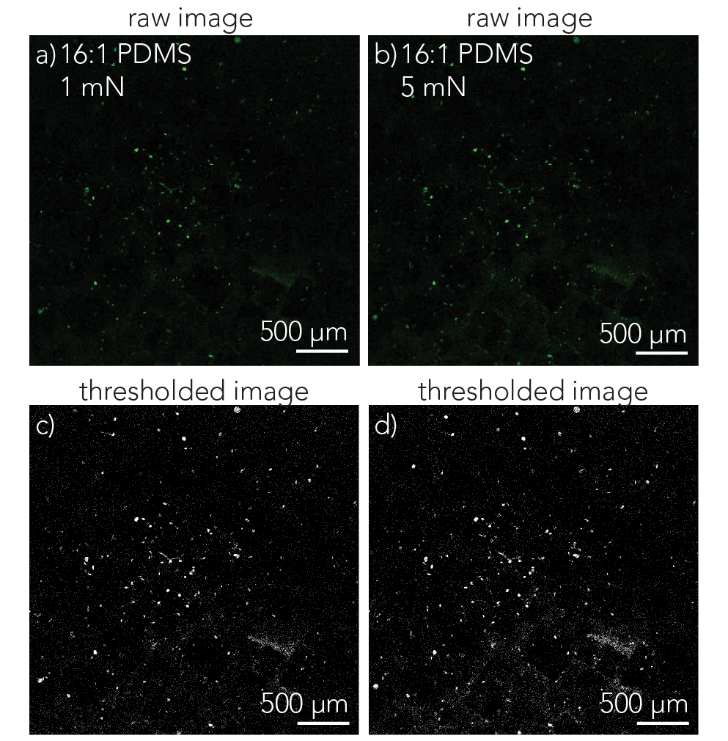


**Fig. S3.** Raw confocal images acquired of fluorescently-labeled, textured 16:1 PDMS probes in contact with a glass countersurface under a normal load, (a) *F_n_* = 1 mN and (b) *F_n_* = 5 mN. Thresholded images of the raw confocal images of this textured 16:1 PDMS under (c) *F_n_* = 1 mN and (d) *F_n_* = 5 mN were used to quantify contact area as a sum of the grey value against the black background.


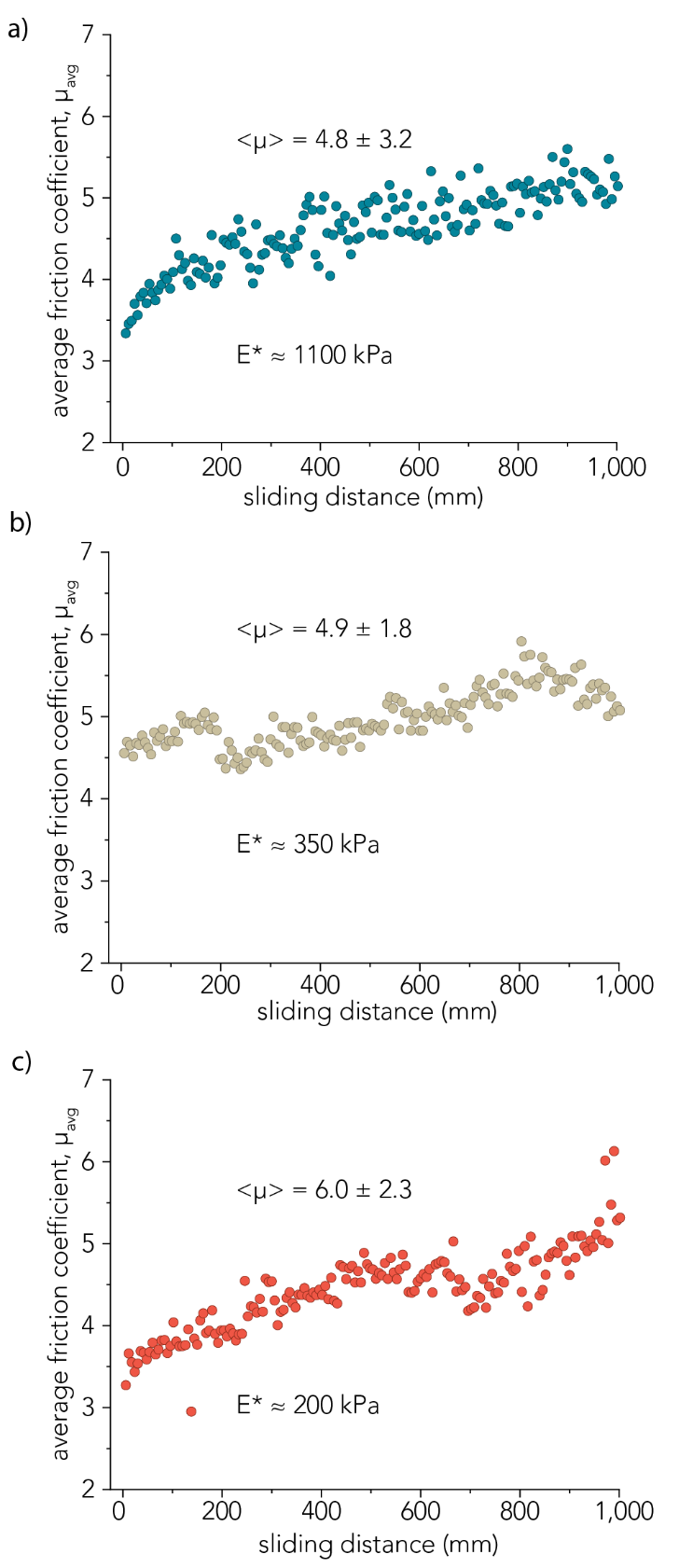


**Fig. S4.** Representative friction coefficient data, *µ_avg_*, are plotted against the sliding distance for (a) a single experiment using textured 10:1 PDMS and (b) a single experiment using textured 12:1 PDMS. The average friction coefficients for the entire sliding experiments, *<µ>*, and the approximate elastic modulus, *E**, are shown on each plot.


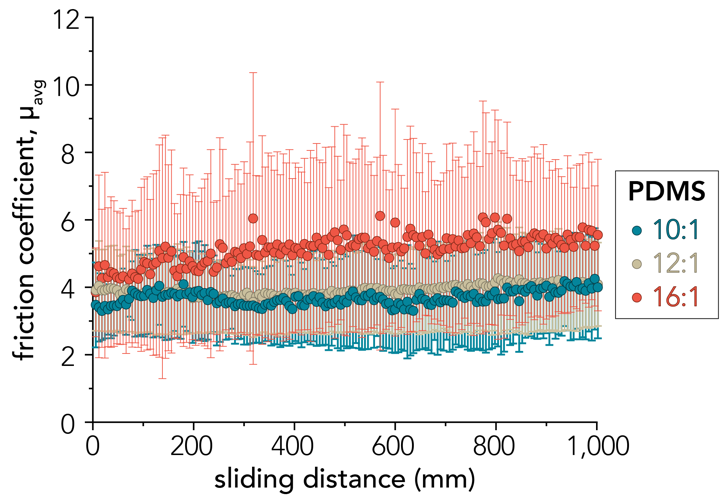


**Fig. S5.** Average friction coefficient, *µ_avg_*, is plotted against sliding distance for 3 different textured PDMS formulations: 10:1 (teal), 12:1 (sand), and 16:1 (coral). The error bars represent the standard deviation in *µ_avg_* across n = 3 unique sliding experiments (n = 4 for 10:1 PDMS).


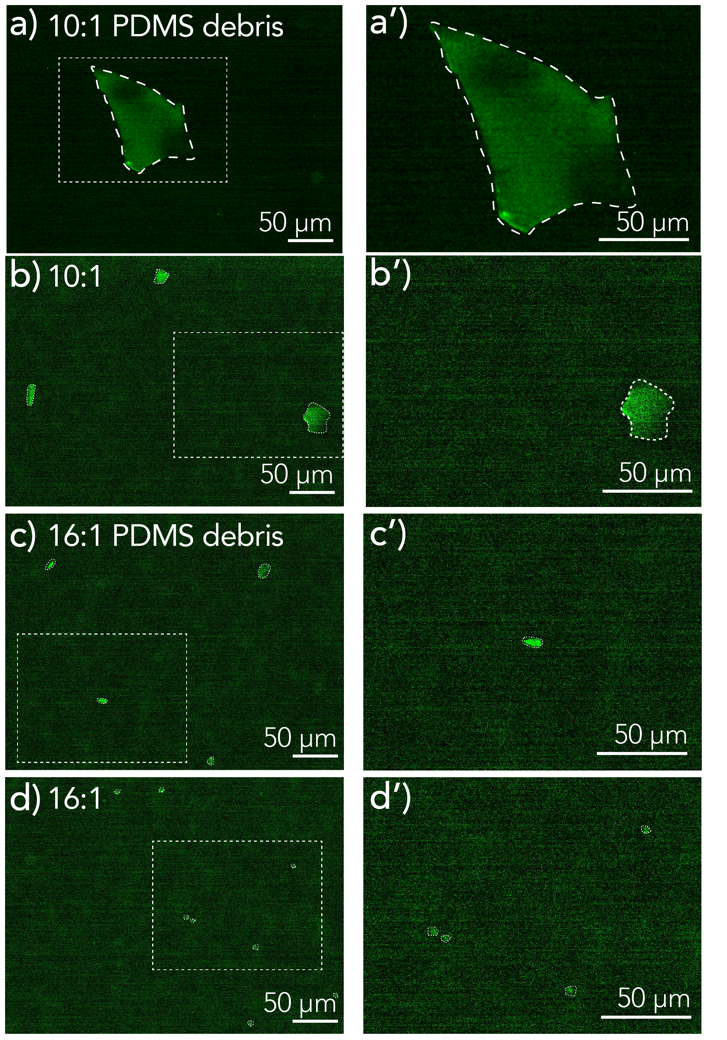


**Fig. S6.** Representative raw confocal images acquired of fluorescently-labeled, textured (a,b) 10:1 PDMS and (c,d) 16:1 PDMS debris particles. Magnified fields of view of these debris particles (a’-d’) were manually traced to yield particle area and aspect ratio.


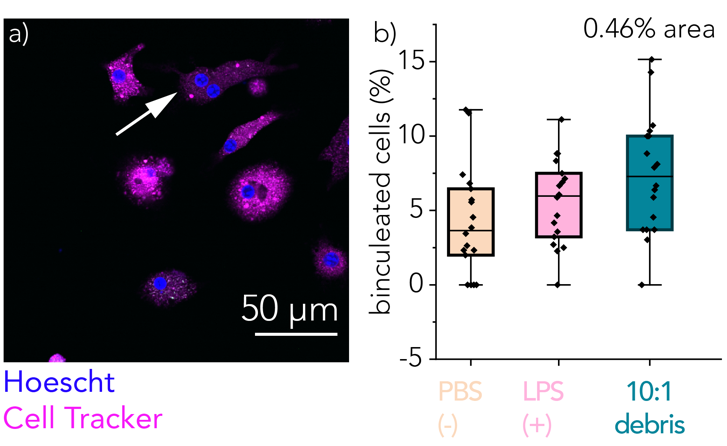


**Fig. S7.** (a) Representative confocal image of a binucleated macrophage (blue: Hoescht; magenta: cell tracker). (b) Percentage of binucleated macrophages are shown for PBS (negative control), lipopolysaccharide (positive control), and a sample treated with a high concentration of 10:1 PDMS debris.


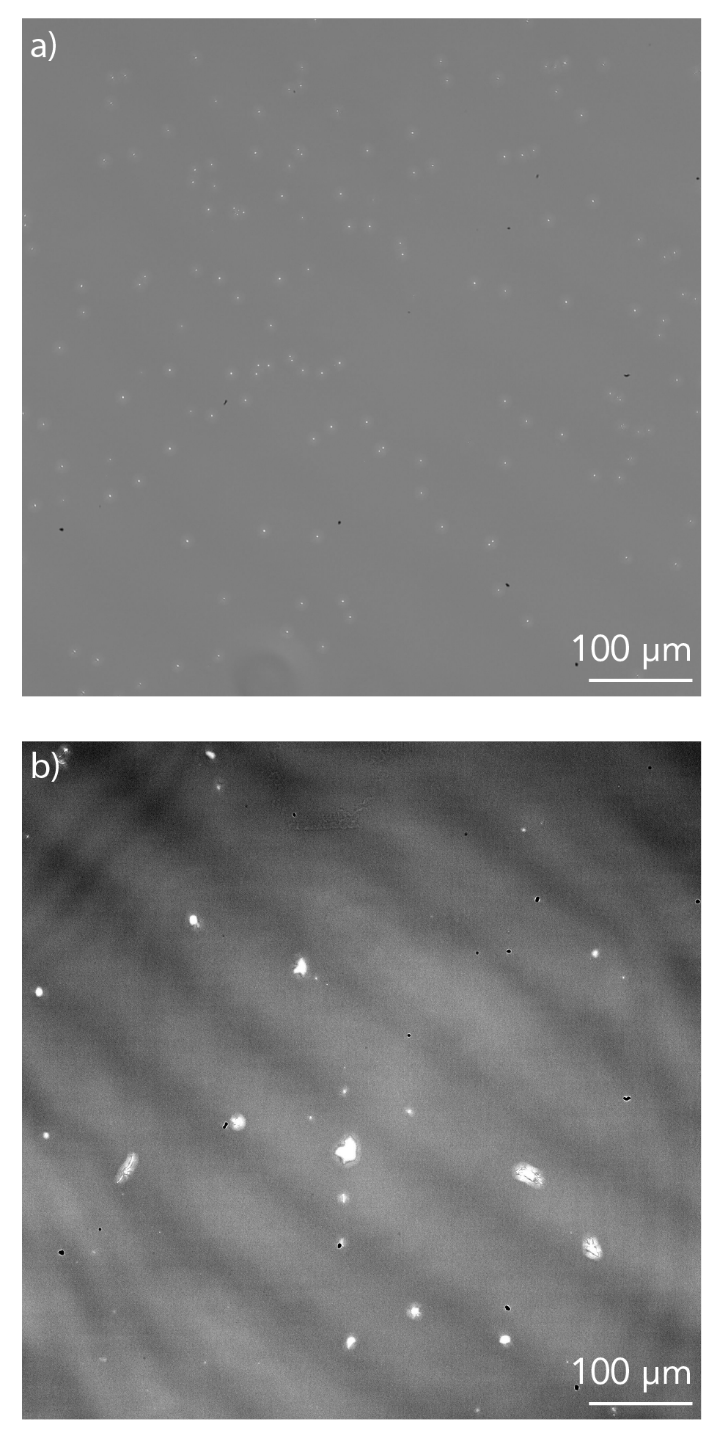


**Fig. S8.** Maximum intensity projections of brightfield images of polyacrylamide particles (a) 1 – 10 µm and (b) 15-25 µm in size that have an elastic modulus, *E**, of approximately 1 kPa, several orders of magnitude softer than the PDMS debris particles.


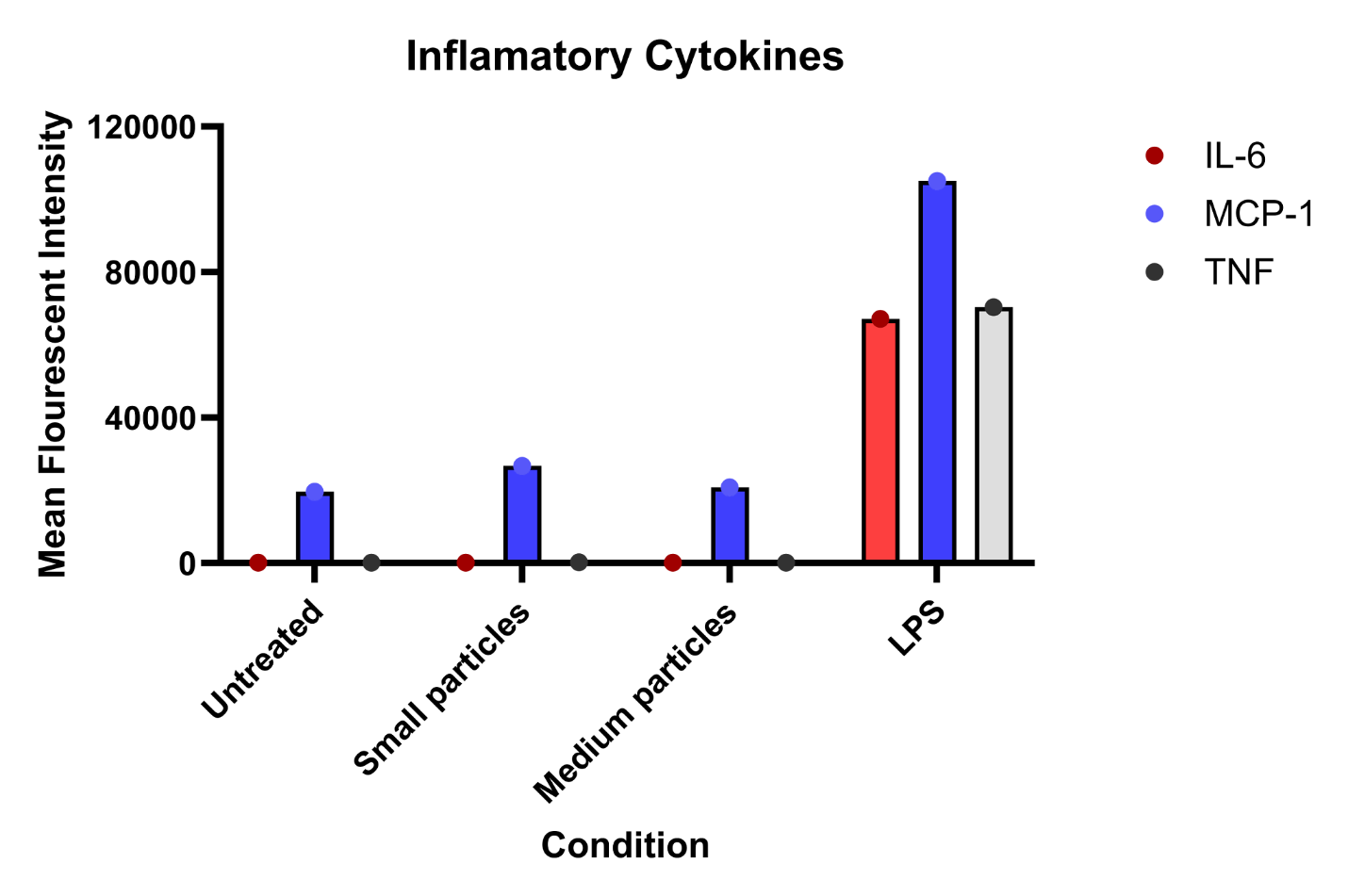


**Fig. S9.** Inflammatory cytokine production by BMDMs following treatment with PBS (untreated), small polyacrylamide particles of 1 -10 µm, medium polyacrylamide particles of 15-25 µm in size, and LPS (+ control).


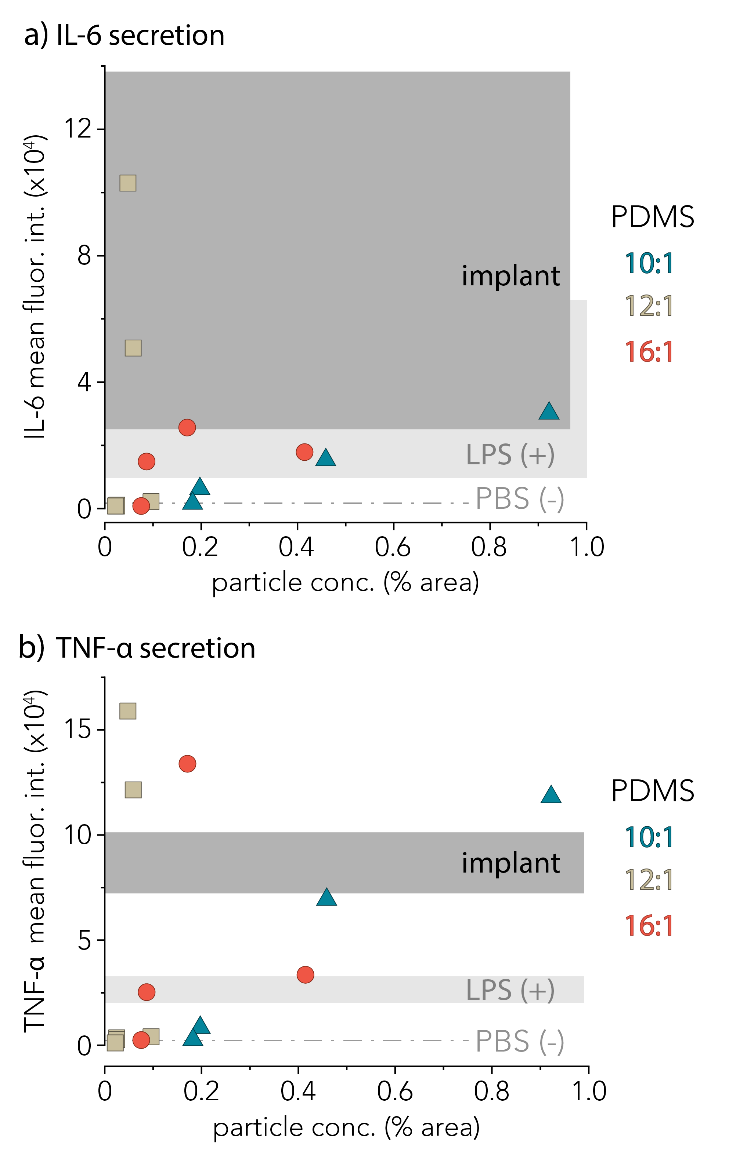


**Fig. S10.** Inflammatory cytokine production (showing the same data as Figure 6) by BMDMs following treatment with PBS (untreated), LPS (positive control), and PDMS debris particles plotted on linear-linear axes.
